# Handwashing in 51 Countries: Analysis of Proxy Measures of Handwashing Behavior in Multiple Indicator Cluster Surveys and Demographic and Health Surveys, 2010–2013

**DOI:** 10.4269/ajtmh.16-0445

**Published:** 2017-06-12

**Authors:** Swapna Kumar, Libbet Loughnan, Rolf Luyendijk, Orlando Hernandez, Merri Weinger, Fred Arnold, Pavani K. Ram

**Affiliations:** 1 University at Buffalo, Buffalo, New York;; 2 Consultant Advisor, World Bank, Washington, District of Columbia;; 3 United Nations Children’s Fund (UNICEF), Joint Monitoring Programme, New York, New York;; 4 International Business and Technical Consultants, Inc. (IBTCI) Global Health Practice, Washington, District of Columbia;; 5 U.S. Agency for International Development, Bureau for Global Health, Washington, District of Columbia;; 6 ICF, Demographic and Health Surveys Program, Fairfax, Virginia

## Abstract

In 2009, a common set of questions addressing handwashing behavior was introduced into nationally representative Demographic and Health Surveys (DHS) and Multiple Indicator Cluster Surveys (MICS), providing large amounts of comparable data from numerous countries worldwide. The objective of this analysis is to describe global handwashing patterns using two proxy indicators for handwashing behavior from 51 DHS and MICS surveys conducted in 2010–2013: availability of soap anywhere in the dwelling and access to a handwashing place with soap and water. Data were also examined across geographic regions, wealth quintiles, and rural versus urban settings. We found large disparities for both indicators across regions, and even among countries within the same World Health Organization region. Within countries, households in lower wealth quintiles and in rural areas were less likely to have soap anywhere in the dwelling and at designated handwashing locations than households in higher wealth quintiles and urban areas. In addition, disparities existed among various geographic regions within countries. This analysis demonstrates the need to promote access to handwashing materials and placement at handwashing locations in the dwelling, particularly in poorer, rural areas where children are more vulnerable to handwashing-preventable syndromes such as pneumonia and diarrhea.

## RATIONALE

Pneumonia and diarrheal disease are leading causes of postneonatal child mortality, accounting for approximately 1.6 million child deaths worldwide in 2013.^1,2^ Even as mortality due to both diseases has been declining, morbidity remains high, with an estimated 1.7 billion episodes of diarrhea and 120 million episodes of pneumonia among children less than 5 years old in 2010.^3^ These illnesses lead not only to preventable mortality but also to health-care seeking, financial costs for families, and lost caloric intake for children. Repeated episodes of diarrhea have been associated with poor growth outcomes and neurocognitive deficits.^4–6^ Recent evidence suggests that children living in households with relatively poor environmental conditions are more likely to have signs consistent with environmental enteropathy and growth faltering.^7^ Handwashing with soap can reduce the risk of diarrhea episodes by 30–47% and respiratory infections by 23%.^8–12^ The available evidence using structured observation methods in households in several low- and middle-income countries suggests that handwashing behavior must be improved substantially.^13,14^ However, in most countries where high child morbidity results from handwashing-preventable infections, there is little data on handwashing behavior. Though studies of household handwashing behavior have been conducted sporadically worldwide, there has been no systematic method of data collection to allow for comparison of handwashing behavior across regions, and identification of national and subnational populations where child mortality and morbidity remain high and where particular need exists with respect to handwashing promotion.^15^ Without meaningful and globally comparable data, it is difficult for governments and international organizations to prioritize handwashing as a public health tool.

The Multiple Indicator Cluster Surveys (MICS) and Demographic and Health Surveys (DHS) are nationally representative household surveys supported by United Nations Children’s Fund, U.S. Agency for International Development, other development partners, and national governments in low- and middle-income countries. Administered about every 3–5 years in more than 100 countries, MICS and DHS collect information on a variety of indicators related to health, population, and nutrition. The use of core questionnaires allows for comparable data on such indicators across numerous countries and over serial surveys.^16,17^ In surveys from the initial rounds (starting in 1985 for DHS and 1995 for MICS) questions on household indicators of handwashing were sporadically included in the core questionnaire, but these questions were not consistently included with standardized wording in every survey until 2009. Also, prior to 2009, questions addressed a range of self-reported and proxy measures, which were worded inconsistently, making it difficult to make comparisons regarding handwashing behaviors across countries and over time.^18^

Measurement of handwashing behavior by self-report risks substantial overestimation of the behavior,^19–22^ and direct measurement of behavior by structured observation is infeasible in large surveys such as MICS and DHS because of the personnel time required.^23^ The available evidence suggests that the presence of a fixed place for washing hands with water and soap present at that place is a feasibly collected measure to describe handwashing behavior in large, nationally representative household surveys such as MICS or DHS.^23–28^ Members of households with soap and water available in a visible, convenient handwashing location wash their hands more frequently than those without handwashing materials at fixed locations.^24^

In 2009, a common set of questions was introduced to the core MICS and DHS questionnaires to document indicators of handwashing behavior: availability of soap anywhere in the dwelling (MICS), and access to water and soap at a place for handwashing (MICS and DHS). With the introduction of a standard set of handwashing questions in the MICS and DHS core questionnaires, we have for the first time a large set of comparable data on handwashing from numerous countries. Routine reporting from both survey networks includes disaggregation of data by potential sources of disparity and geographic regions within countries, allowing novel insight into differences affecting handwashing behavior and revealing potential targets for intensive handwashing promotion. Using data from 51 MICS and DHS surveys with comparable handwashing questions administered between 2010 and 2013, we describe global patterns in handwashing using two indicators: availability of soap anywhere in the dwelling and access to a handwashing place with soap and water. We describe handwashing across geographic regions, wealth quintiles, and rural and urban settings.

## METHODS

In household MICS and DHS surveys, interviewers recorded the presence of soap and water at a place for handwashing by asking the respondent to “Please show me where members of your household most often wash their hands.” For households in which a place for handwashing was observed, interviewers recorded the presence of soap (soap or detergent in a bar, liquid, powder, or paste form), other cleansing agents (such as ash, mud, or sand), and water. If a handwashing place could not be observed, interviewers recorded the reason for the nonobservation, including whether the household refused permission to observe the place. In households in which soap was not observed at a place for handwashing, interviewers in MICS surveys assessed the availability of soap anywhere in the dwelling by asking the respondent “Do you have any soap or detergent (or other locally used cleansing agent) in your household for washing hands?” If yes, the respondent was asked to show the soap to the interviewer.

Both MICS and DHS provide standard tabulation plans for key indicators (DHS: http://dhsprogram.com/publications/publication-dhsm6-dhs-questionnaires-and-manuals.cfm; MICS: http://mics.unicef.org/tools#analysis). Handwashing-related data from MICS and DHS surveys conducted between 2010 and 2013 were compiled by UNICEF from published survey reports that applied the standard tabulation plans for those indicators. For the availability of soap anywhere in the dwelling, the proportion was calculated based on the entire set of households included in the sample. For the indicator of soap and water at the handwashing location, the standard tabulation plan excludes from the analysis households in which the respondent denied permission to see the place where household members wash their hands, households which do not have one specific place where members wash their hands, and households which have no handwashing place. For the analysis presented in this paper, we included in the denominator all households irrespective of whether the household had an observed handwashing location to portray a more representative picture of overall access to handwashing facilities, since some households simply did not have observable handwashing locations (Table 1).

**Table 1:** Observation of handwashing materials, as measured in MICS/DHS data by World Health Organization region, 2010–2013

Country	Year*	Survey type	Total HH	HH with soap† anywhere in the dwelling‡ (%)	Households with soap and water at observed HW place (%)
Benin	2012	DHS	17,422		9.4
Burkina Faso	2010	DHS	14,424		10.1
Burundi	2010	DHS	8,596		5.1
CAR	2010	MICS	11,756	77.4	14.4
Chad	2010	MICS	16,386	55.0	22.3
Cote d’Ivoire	2012¥	DHS	9,686		13.0
DRC	2010	MICS	11,393	50.1	3.8
Equatorial Guinea	2011	DHS	4,223		22.7
Ethiopia	2011	DHS	16,702		0.0
Gambia	2010	MICS	7,791	55.0	11.0
Ghana	2011	MICS	11,925	63.6	11.9
Guinea	2012	DHS	7,109		7.0
Guinea-Bissau	2010	MICS	9,859		3.4
Kenya, Nyanza	2011	MICS	6,828	85.2	2.6
Madagascar, south	2012	MICS	2,968	33.0	3.7
Malawi	2010	DHS	24,825		0.2
Mozambique	2011	DHS	13,919		10.8
Nigeria	2011	MICS	29,077	61.5	13.2
Rwanda	2010	DHS	12,540		2.1
Senegal	2011¥	DHS-MICS	7,902	20.8	20.2
Sierra Leone	2010	MICS	11,394	40.9	12.3
Swaziland	2010	MICS	4,834	88.8	34.7
Togo	2010	MICS	6,039	65.4	10.2
Uganda	2011	DHS	9,033		2.3
Zimbabwe	2010	DHS	9,756		24.7
Afghanistan	2011¥	MICS	13,116	74.4	42.6
Iraq	2011	MICS	35,701	99.1	91.5
Pakistan	2013¥	DHS	12,943		54.0
Pakistan, Balochistan	2010	MICS	11,612	72.0	41.2
Pakistan, Punjab	2011	MICS	95,238	94.6	74.1
Tunisia	2012	MICS	9,171	95.5	78.1
Bangladesh	2011	DHS	17,141		21.4
Bhutan	2010	MICS	14,676	98.9	78.7
Indonesia	2012	DHS	43,852		73.6
Nepal, mid/far western	2010	MICS	5,899	68.0	39.8
Nepal	2011	DHS	10,826		47.7
Armenia	2010	DHS	6,700		84.5
Bosnia/Herzegovina	2012¥	MICS	5,778	98.6	95.6
Bos/Herz, Roma	2012¥	MICS	1,544	96.5	86.5
Kyrgyz Republic	2012	DHS	8,040		85.2
Serbia	2010	MICS	6,392	99.1	96.4
Serbia, Roma	2010	MICS	1,711	95.7	85.1
Tajikistan	2012	DHS	6,432		72.6
Cambodia	2010	DHS	15,667		50.7
Mongolia	2010	MICS	10,092	98.9	92.1
Vietnam	2011¥	MICS	11,614	95.1	84.7
Belize	2011	MICS	4,424	93.2	71.7
Costa Rica	2011	MICS	5,561	91.0	72.5
Haiti	2012	DHS	13,181		21.5
Honduras	2012¥	DHS	21,362		79.3
Suriname	2010	MICS	7,407	96.2	63.4

DHS = Demographic and Health Surveys; MICS = Multiple Indicator Cluster Surveys; HH = household; HW = handwashing.

* Year of completion.

† Includes detergent and other locally used cleansing agents.

‡ Indicator only assessed in MICS questionnaires, with the exception of the Guinea-Bissau MICS.

We categorized countries that implemented a MICS or DHS survey by World Health Organization (WHO) region: Africa Region, Eastern Mediterranean Region, southeast Asia Region, European Region, Western Pacific Region, and the Region of the Americas.

We describe each indicator at the individual country level and by WHO region to understand differences within broader geographic areas. We also provide information on each indicator according to the following potential sources of disparity within each survey: wealth quintile, area (urban/rural). Among nationally representative surveys, we evaluated geographic disparities (e.g., between provinces within a country). As part of the standard analyses by the DHS and MICS programs, a wealth index was created for households within each survey dataset using data on household ownership of goods (e.g., bicycles, televisions), housing characteristics (e.g., material of wall/floor/roof), and basic services (e.g., type of water source or sanitation system).^29^ Wealth indices were used to categorize households within each survey population to wealth quintiles.

Disaggregated data are not presented in the figures for countries in which fewer than 10% of households overall had soap and water at a handwashing location due to lack of heterogeneity in the total sample (Figures 3A–C and 4A–C).

## RESULTS

Between 2010 and 2013, 28 MICS and 23 DHS with standard handwashing indicators became available for analysis. Of the 51 surveys, three were from the Western Pacific Region, five from the southeast Asian Region, six from the Eastern Mediterranean Region, seven from the European Region, 25 from the Africa Region, and five from the Region of the Americas. We identified seven surveys that were conducted among subpopulations and, thus, were not nationally representative: the Roma population of Serbia (Serbia, Roma), the Roma population of Bosnia and Herzegovina (Bosnia/Herzegovina, Roma), south Madagascar (Madagascar, south), the Nyanza Province of Kenya (Kenya, Nyanza), mid and far western Nepal (Nepal, mid and far western), the Balochistan Region of Pakistan (Pakistan, Balochistan), and the Punjab Region of Pakistan (Pakistan, Punjab). The total number of households in nationally representative surveys ranged from 4,223 in Equatorial Guinea to 43,852 in Indonesia, whereas the total number of households in subpopulation surveys ranged from 1,544 in the Roma population of Bosnia and Herzegovina to 95,238 in the Punjab Region of Pakistan (Table 1).

For households with a handwashing place observed by the interviewer, information was collected on whether soap and water were available at that place (Table 1). In several countries, a high percentage of households in which a handwashing location was not observed had handwashing locations outside the compound (e.g., 96.3% in Ethiopia) or handwashing locations that were unobserved for “other reasons” (e.g., 53.5% in Rwanda) (Supplemental Table 1).

**Figure 1.**
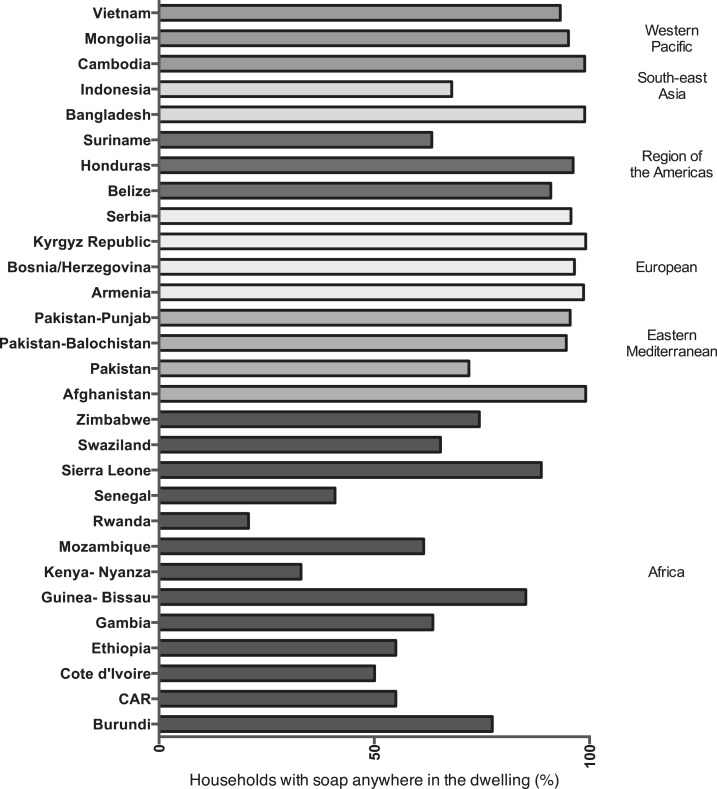
Percentage of households observed to have soap for handwashing anywhere in the dwelling, Multiple Indicator Cluster Surveys, 2010–2013.

In MICS surveys that included information on whether soap was available anywhere in the dwelling (all except Guinea-Bissau), availability of soap in the household ranged from 20.8% in Senegal to 99.1% in Iraq and Serbia (Table 1, Figure 1). Eleven other countries had levels of soap availability exceeding 90%.

**Figure 2.**
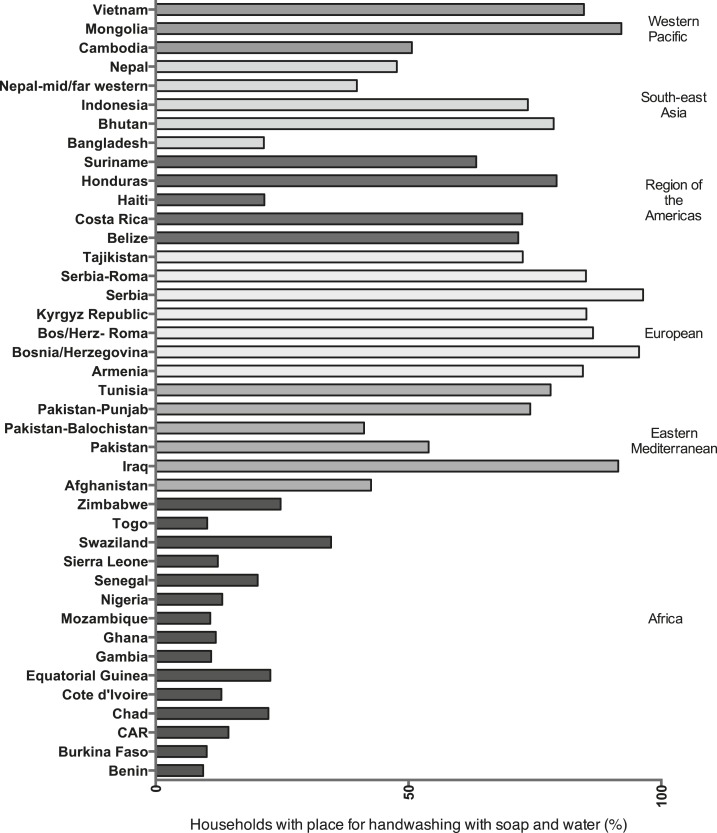
Percentage of households observed to have handwashing place with soap and water, Multiple Indicator Cluster Surveys and Demographic and Health Surveys, 2010–2013, by World Health Organization Region.

Across all surveys, Serbia had the highest proportion of households surveyed with soap and water at a handwashing place (96.4%) and Ethiopia had the lowest (less than 0.1%) (Table 1, Figure 2). In four countries, more than 90% of households with an observed handwashing place had soap and water and in 11 countries, fewer than 10% of households did so. In Africa, the proportion of all households with soap and water at an observed handwashing place was low, ranging from less than 0.1% in Ethiopia to 34.7% in Swaziland (Table 1, Figure 4). Availability of soap and water at a handwashing place was substantially higher in the Eastern Mediterranean Region, ranging from 42.6% in Afghanistan to 91.5% in Iraq in nationally representative surveys. In the southeast Asia Region, 78.7% of households in Bhutan had handwashing places with soap and water, compared with 21.4% in Bangladesh. With substantially lower rates of soap and water at a handwashing place than other countries in their respective regions, Cambodia and Haiti were clear regional outliers.

**Figure 3.**
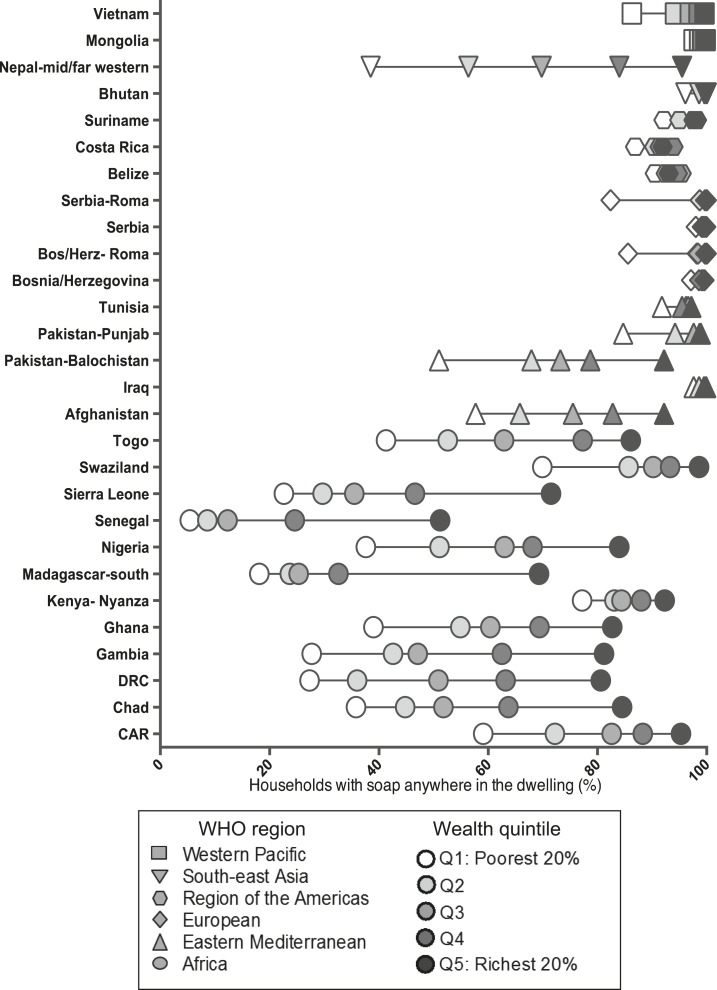

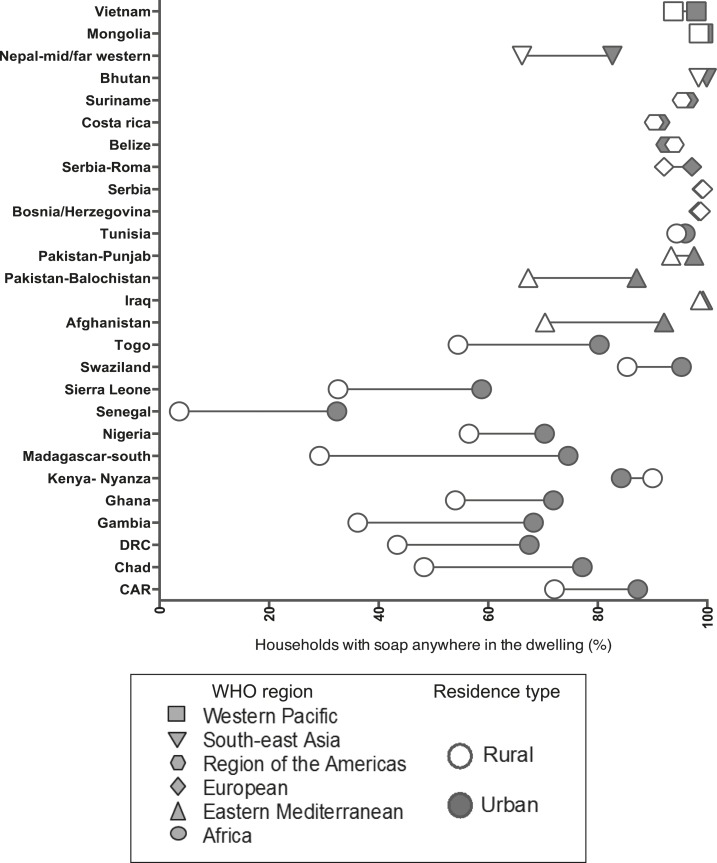

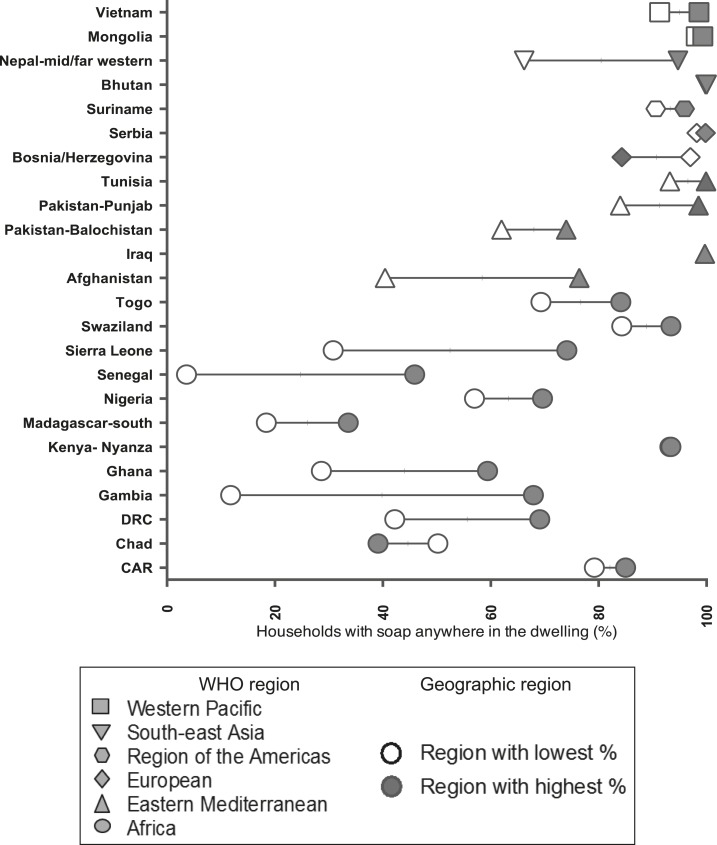
Percentage of households observed to have soap for handwashing anywhere in the dwelling, (**A**) by wealth quintile, (**B**) by residence type, and (**C**) by geographic region. Multiple Indicator Cluster Surveys, 2010–2013.

**Figure 4.**
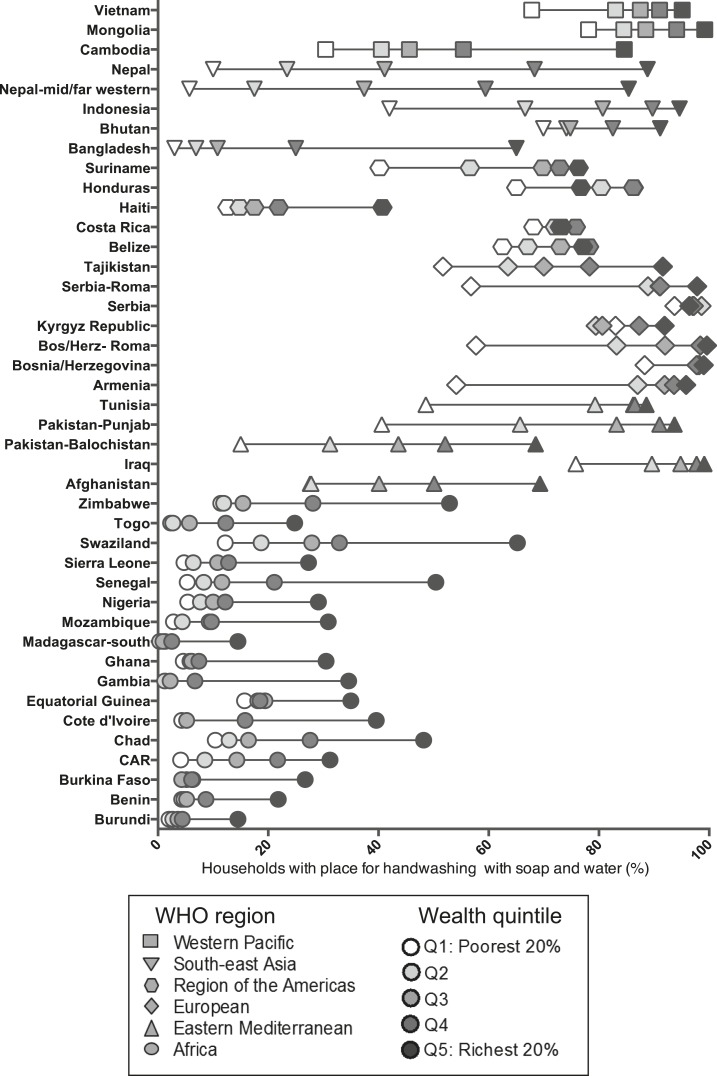

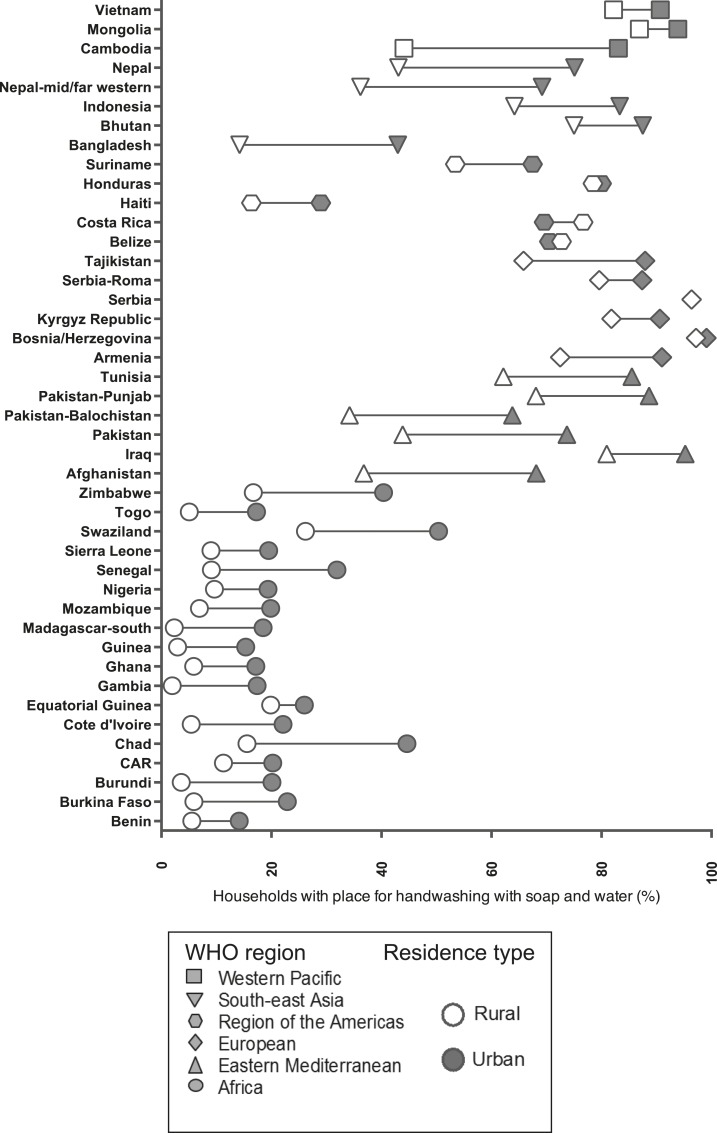

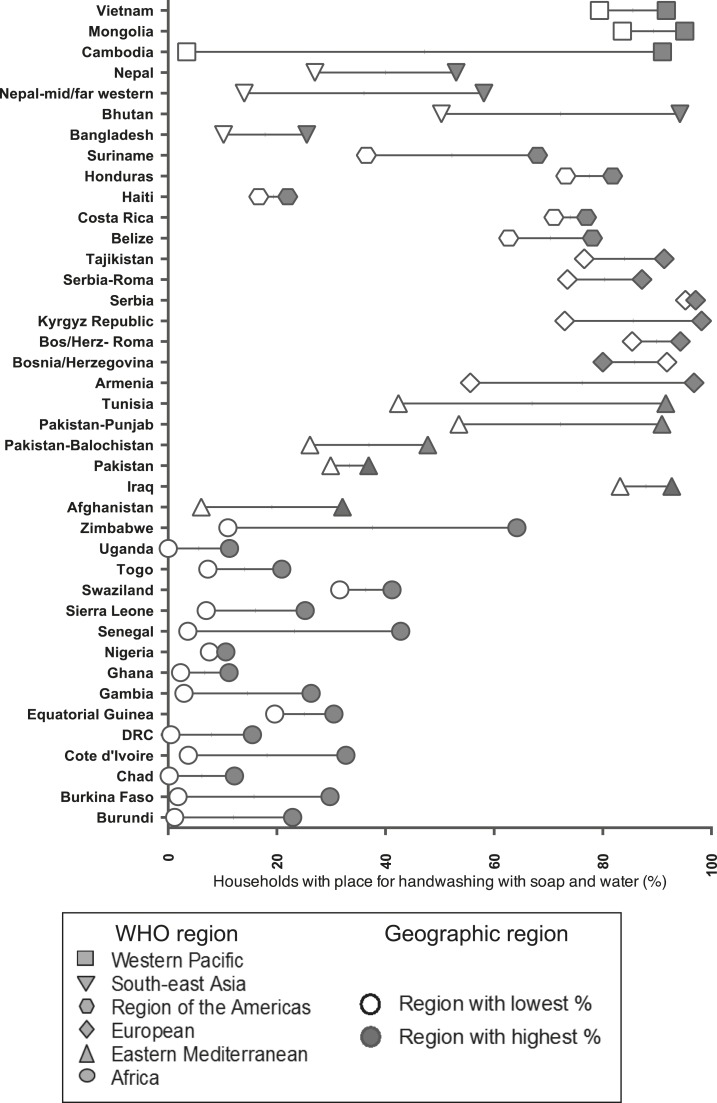
(**A**) Percentage of households observed to have handwashing place with soap and water, by wealth quintile, Multiple Indicator Cluster Surveys (MICS) and Demographic and Health Surveys (DHS), 2010–2013. (**B**) Percentage of households observed to have handwashing place with soap and water, by residence type, MICS and DHS, 2009–2013. (**C**) Geographic regions with highest and lowest percentage of households observed to have handwashing place with soap and water, MICS and DHS, 2009–2013.

In almost every country, households in higher wealth quintiles were much more likely to have soap in the dwelling and places for handwashing with soap and water than those in lower wealth quintiles (Figures 3A and 4A), but the differences were small or nonexistent in the countries in which availability of soap is almost universal. For example, in the mid- and far-western regions of Nepal, households in the richest quintile were 2.5 times as likely to have soap in the dwelling as households in the poorest quintile (95.5% versus 38.5%). However, there was almost universal coverage in the nationally representative survey of Serbia with little difference across the wealth quintile. Similarly, in the 2011 Nepal-mid and far western MICS, there was a difference of almost 80% points in households with soap and water at a handwashing place between households in the highest and lowest wealth quintiles (5.7% versus 85.4%). The Roma population in Serbia had a variation of 41% points between the highest and lowest wealth quintiles.

Within countries, households in urban areas were much more likely to have soap in the dwelling and a handwashing place with soap and water than households in rural areas (Figure 3B and 4B). Nearly 30% of rural households in Madagascar-south had soap available in the household compared with 74.6% of urban households (Figure 3B and 4B). In Cambodia, 83.1% of urban households were observed to have soap and water at a handwashing place, compared with only 44.1% of rural households.

The Gambia has the greatest disparity between geographic regions within a single country; 11.8% of households in the Kuntaur Region of the country were found to have soap anywhere in the household compared with 67.9% of households in the West Region (Figure 3C and 4C). Cambodia had the largest within-country geographic disparities in access to handwashing places with materials: 3.4% in the Takeo Region compared with 91.0% in the Phnom Penh Region.

There were also differences in disparities among countries within the same WHO region. For example, within the Western Pacific Region, the disparity in access to a handwashing place with soap and water across wealth quintiles was more evident in Cambodia (30.4–84.6%) than in Mongolia (78.1–99.2%) (Table 1). Similarly, the rural–urban disparities were more pronounced in Cambodia (rural: 44.1%, urban: 83.1%) than in Mongolia (rural: 86.9%, urban: 93.9%) (Figures 3B and 4B).

## DISCUSSION

Our analyses of proxy measures of handwashing from 51 DHS and MICS household surveys, together with information from an analysis of 42 studies demonstrating that only 19% of people wash their hands after fecal contact when observed,^14^ highlight the need to improve handwashing with soap globally to reduce the continued high burden of diarrhea and pneumonia morbidity and mortality. The overwhelming majority of under-five deaths occur in low- and middle-income countries, with about three-quarters of all child deaths occurring in sub-Saharan Africa and South Asia.^30^ Under-five mortality is highest in rural areas and among poorer and less educated communities.^31^ In particular, children in sub-Saharan Africa are nearly 14 times as likely to die before the age of five as children in high-income countries.^30^ A sizable proportion of these deaths are due to preventable causes including diarrhea and pneumonia, both of which can be reduced by handwashing with soap.^1^ The DHS and MICS surveys indicate that soap for handwashing is least likely to be found in households in the Africa Region. Moreover, fixed handwashing places with soap and water were infrequently observed in most African countries, along with some countries in southeast Asia, Western Pacific, and the Americas, indicating that promotion of handwashing as a preventive measure is not only necessary for lower-income countries, but also in higher-income countries where disparities still exist and could also have adverse sequelae.

Availability of soap for handwashing in the dwelling is a crude indicator of handwashing behavior, since the sheer presence of soap in a household does not mean that its residents actually wash their hands with that soap. In addition, since the MICS survey enumerators asked specifically to observe soap for handwashing, it is possible that households may have had soap available for purposes such as laundry and dishwashing but they have not prioritized its use for handwashing, or identified it as such. However, handwashing with soap is not possible if the material is entirely absent from the dwelling. The poorest households, and those in rural areas, may not have soap for handwashing for a variety of reasons, including cost of materials, access to materials in local shops and markets, and dynamic of decision-making power within household members that may determine purchase of soap and other health-related goods above other expenditures. Distribution of commercial goods to rural areas with poor road networks may be challenging and, thus, rural households might be less able to purchase soap for handwashing than those in urban areas. Additionally, rural regions may be not prioritized and given equal consideration by the companies controlling handwashing materials.

Low- or no-cost hand cleansing agents, such as ash, were rarely observed in households in any of the countries included in this analysis. Ash has been shown to be microbiologically effective in removing organisms from hands, although there are no data supporting or refuting the impact of ash use for handwashing on health outcomes.^32^

Increasing the availability and promotion of affordable alternatives to bar soap, such as soapy water,^33^ may close the gap in access to soap for handwashing in low-income households. The ease of soapy water preparation, its low cost, and equivalent antimicrobial efficacy may facilitate uptake, especially since these materials may be less likely to be stolen than bar soap.^33^ Soapy water is used at relatively small scale currently but should be considered for possible inclusion as a type of soap in future administrations of MICS and DHS.^33,34^ In addition, social marketing approaches with public–private partnerships and hygiene promotion may be useful to generate demand and increase affordability within the areas of greatest need.^35^ Successful behavior change interventions have yielded increases in handwashing behavior at the promoted times without the specific promotion of maintenance of handwashing materials^36^ at designated locations; however, adherence to the promoted behavior is predicated on having the materials available in the home.^35,37^

Households may maintain a fixed location for handwashing and ensure the presence of soap and water at that location because they prioritize handwashing, or household members may wash their hands more frequently as a result of those materials being visible or conveniently located where and when they are needed, as evidenced by qualitative research in numerous countries.^13,38^ The data supporting this indicator as a marker of handwashing behavior are largely observational in nature; multiple studies to validate proxies of handwashing behavior have shown that presence of water, soap, or both materials at the handwashing place were associated with observed increased handwashing behavior.^23,24^ The presence of water and a handwashing place was associated with a decrease in respiratory illness outcomes in one study in Bangladesh.^27^ Also in Bangladesh, better hand cleanliness was observed among members of households with soap available in the handwashing place used after defecation.^28^ In an analysis of similarly collected data on various self-reported and proxy measures of handwashing from Peru, Senegal, and Vietnam, the only indicator associated with observed handwashing with soap during structured observation in all three countries was the presence of soap and water at a handwashing location.^24^ However, promotion of maintenance of a handwashing station with materials may not result in substantial gains in handwashing behavior in the absence of other behavior change communication (S. Ashraf, personal communication). Several randomized controlled trials of interventions combining the provision of handwashing materials with behavior change communication, promoting maintenance of materials at a fixed place along with handwashing with soapy water after defecation and before food preparation, are underway in Bangladesh and Kenya, and they should be informative regarding the behavioral and health effects of such a comprehensive behavior change approach.^39^

The MICS and DHS data demonstrate broad global disparities in the maintenance of a fixed handwashing location with the materials needed to support handwashing. Although such fixed and fully stocked handwashing locations were found nearly universally in dwellings in most of the survey countries in the Eastern Mediterranean and European Regions, they were rarely confirmed in dwellings in most African countries included in the analysis. Similar to the general pattern shown here for Africa, Kamm and others found that fewer than 1% of households in Western Kenya were observed to have soap and water at a fixed location.^40^ In our experience, in some countries in Africa, many households rely on mobile devices for handwashing. When hands need to be washed, the individual may move a jug, basin, and soap from inside the home to the outdoor courtyard to wash hands. There is no published information on the frequency of such mobile device use, nor is there information on whether the concepts of convenience and visual cueing operate differently in the societies in which the social norm is to keep handwashing materials at fixed locations where hands are washed. But given that the countries in which these fully stocked fixed handwashing locations were infrequently found also share high rates of child mortality from handwashing-preventable diseases such as diarrhea and pneumonia, gaining further understanding of the physical and social norm-related barriers to handwashing with soap in Africa is vital.

The handwashing indicators now included in the MICS and DHS describe household-level factors. Neither informs us directly about the behavior of individuals and when that behavior is practiced. From these existing indicators, we cannot know whether soap maintained in the home or at a fixed handwashing location is used for handwashing by the relatively healthy adolescent or by the mother of a particularly vulnerable neonate. From the household survey data, we cannot elucidate within-household disparities nor can we assess factors that may prevent some members of the household, for example, young children or the disabled, from accessing soap for handwashing. Structured observations can directly reveal the behaviors of individuals without proxy measures and describe within-household disparities, which are largely unstudied. But observation of handwashing behavior over multiple hours is simply not feasible within the already lengthy data collection involved in the MICS and DHSs.^19–23^

Our study has a few limitations. First, if the handwashing place was not in the dwelling/plot/yard, it was not observed and no information was collected on the presence of soap and water. Public places for handwashing with soap and water were, thus, not captured, potentially underestimating the availability of fixed handwashing locations. However, a fixed location for handwashing placed at some distance outside the household’s immediate environment could be inconvenient enough to discourage handwashing. Other reasons for lack of observation were not fully delineated in the survey instruments. The proportion of households not observed to have a handwashing station due to these “other reasons” was as high as 53% in Rwanda. Potential explanations could include use of portable devices for handwashing (e.g., a jug and a basin), miscommunication between the interviewer and respondent, and physical constraints (physically unable to observe the location). It is important to conduct confirmatory studies in such settings to understand the limitations of the indicator. For the purposes of this study, we deviated from standard tabulation plans which excluded households where handwashing locations were not observed. This study instead measured proportions out of the total number of households, which provides a more realistic representation of access to handwashing materials in a population, since in some cases, a high proportion of handwashing stations were not observed. Ideally, we recommend all nationally representative surveys include reasons for unobserved handwashing locations to parse out those households that did not have a fixed place for handwashing from households with places to wash hands, but for which permission was not granted for observation.

The individual sources of disparity may be interconnected. The poorest households may also have the least educated heads of household and are more likely to live in rural areas. In this descriptive analysis, we are not adjusting for potential confounding among these various sources of disparity and availability of soap, or observation of soap and water at fixed handwashing locations. Further, estimating wealth principally on the basis of household goods risks masking other sources of disparity; however, the wealth index is universally applied in MICS and DHS analyses and represents a widely recognized metric for evaluating within-country inequities.

## CONCLUSIONS

Handwashing with soap can substantially reduce the prevalence of pneumonia and diarrhea, two leading causes of child morbidity and mortality worldwide. Our analysis of data from 51 recent household surveys worldwide indicates a substantial need to increase availability of soap for handwashing, and to promote placement of soap and water at fixed handwashing places for many households to increase handwashing with soap. The need is particularly pressing among poorer households and households in rural areas where children may be at greatest risk for preventable mortality. However, this analysis also shows that disparities persist even in higher-income countries. The preventive potential of handwashing with soap was not addressed in the Millennium Development Goals, which expired in 2015. With their focus on equity and the incorporation of handwashing into Sustainable Development Goal target 6.2, the post-2015 Sustainable Development Goals have the potential to substantively level the access to preventive measures, such as soap for handwashing, to promote child survival, health, and growth.^41^

## Supplementary Material

Supplemental Table.
