# Supplementary material for: Handwashing in 51 Countries: Analysis of Proxy Measures of Handwashing Behavior in Multiple Indicator Cluster Surveys and Demographic and Health Surveys, 2010–2013
Source: Am J Trop Med Hyg. 2017 Jun 12;97(2):447–59. doi: 10.4269/ajtmh.16-0445 (PMC5544068; doi:10.4269/ajtmh.16-0445)
Supplement: Supplementary file 1 [file tpmd160445.SD1.pdf]

Supplemental Table 1

Explanations for lack of observation of handwashing locations in household surveys, MICS/DHS data by World Health Organization region, 2010–2013*†

Country	Year	Survey type	Households without a place for handwashing in the dwelling/plot/yard (%)	Households where permission was not granted to observe handwashing place (%)	Handwashing place not observed due to other reasons (%)	Missing
Africa Region						
Burkina Faso	2010	DHS	5.8	0.4	18.6	0.0
Burundi	2010	DHS	6.4	0.4	1.2	0.0
DRC	2010	MICS	73.6	2.0	9.9	0.0
Ethiopia	2011	DHS	96.3	0.2	1.3	0.0
Ghana	2011	MICS	70.3	0.4	5.5	NA
Kenya–Nyanza	2011	MICS	95.7	0.2	NA	0.0
Madagascar - south	2012	MICS	17.8	0.3	0.3	NA
Malawi	2010	DHS	90.9	0.4	0.7	0.0
Nigeria	2011	MICS	56.5	9.3	10.3	0.0
Rwanda	2010	DHS	35.8	0.3	53.5	0.0
Senegal	2011‡	DHS-MICS	46.7	1.7	6.4	0.0
Sierra Leone	2010	MICS	28.2	2.2	3.7	0.2
Swaziland	2010	MICS	20.1	3.7	2.3	0.1
Togo	2010	MICS	53.9	2.1	18.4	0.2
Uganda	2011	DHS	54.2	1.9	14.9	0.0
Zimbabwe	2010	DHS	7.6	2.6	33.4	0.0
Eastern Mediterranean Region						
Afghanistan	2011‡	MICS	33.3	4.3	2.1	0.1
Iraq	2011	MICS	3.8	0.5	0.9	0.0
Pakistan-Balochistan	2010	MICS	21.4	7.3	4.4	0.4
Pakistan-Punjab	2011	MICS	1.0	1.7	0.6	0.0
Tunisia	2012‡	MICS	5.2	9.2	0.4	0.1
South-East Asia Region						
Bhutan	2010	MICS	1.9	0.1	0.4	0.0
Nepal-mid and far western	2010	MICS	5.3	0.3	0.6	0.0
Nepal	2011	DHS	0.1	0.1	0.1	0.0
European Region						
Armenia	2010	DHS	6.4	1.5	3.7	0.0
Bosnia/Herzegovina	2012‡	MICS	0.9	0.9	0.5	0.0
Bosnia/Herzegovina-Roma	2012‡	MICS	4.0	NA	1.5	0.1
Serbia	2010	MICS	0.7	1.3	0.8	0.1
Serbia-Roma	2010	MICS	2.1	2.5	2.2	0.2
Western Pacific Region						
Cambodia	2010	DHS	32.7	0.1	0.9	0.0
Mongolia	2010	MICS	30.3	0.0	2.9	0.0
Vietnam	2011‡	MICS	0.7	0.9	0.4	0.0
Region of the Americas						
Belize	2011	MICS	7.3	9.9	6.9	NA
Costa Rica	2011	MICS	0.9	14.1	4.6	0.2
Suriname	2010	MICS	11.2	9.6	5.6	0.1

DHS = Demographic and Health Surveys; MICS = Multiple Indicator Cluster Surveys; NA = not applicable.

* This table breaks down the percentage of households which were not observed to have handwashing places for various reasons: Handwashing place was not in the dwelling/plot/yard, no permission was granted to observe handwashing place, other reasons, and missing data on reason.

† MICS surveys without data on unobserved handwashing places: Central African Republic, Chad, Gambia, and Guinea-Bissau. DHS surveys without data on unobserved handwashing places: Mozambique, Benin, Cote d'Ivoire, Equatorial Guinea, Guinea, Bangladesh, Pakistan, Kyrgyz Republic, Tajikistan, Indonesia, Haiti, and Honduras.

‡ Year of completion.
